# Altering the dosage of meiotic crossover-associated RING finger proteins affects crossover number and interference in Drosophila

**DOI:** 10.1093/g3journal/jkag121

**Published:** 2026-05-08

**Authors:** Emerson Frantz, Priscila Santa Rosa, Susan McMahan, Jeff Sekelsky

**Affiliations:** Department of Biology, University of North Carolina at Chapel Hill, Chapel Hill, NC 27599, United States; Curriculum in Genetics and Molecular Biology, University of North Carolina at Chapel Hill, Chapel Hill, NC 27599, United States; Integrative Program for Biological and Genome Sciences, University of North Carolina at Chapel Hill, Chapel Hill, NC 27599, United States; Department of Biology, University of North Carolina at Chapel Hill, Chapel Hill, NC 27599, United States; Integrative Program for Biological and Genome Sciences, University of North Carolina at Chapel Hill, Chapel Hill, NC 27599, United States

**Keywords:** Drosophila, meiotic recombination, RING fingers, Dros2026

## Abstract

Crossovers play a critical role in ensuring correct reductional segregation of homologous chromosomes in the first meiotic division. Crossing over is initiated by formation of DNA double-strand breaks (DSBs), but the number of DSBs is greater than the number of crossovers. Which recombination sites become crossovers, versus being repaired as noncrossovers, is not random, but is subject to several crossover patterning phenomena. One current model for crossover designation proposes that crossover-associated RING finger proteins (CORs) undergo the biophysical process of coarsening, in which larger accumulations continue to get larger, destining those sites to become noncrossovers, and smaller accumulations go away, resulting in those sites being repair as noncrossovers. Genetic and cytological studies of the three CORs in *Drosophila melanogaster*, Vilya, Narya, and Nenya, are consistent with this model. We tested another prediction of the coarsening model, that differences in COR dosage will lead to differences in crossover number. In females heterozygous for a deletion of *vilya*, significantly fewer double-crossovers are observed. Conversely, crossovers are elevated in females carrying a duplication of *vilya* and in females coordinately overexpressing all three CORs. These findings lend additional support to the proposal that crossover designation in *D. melanogaster* occurs through coarsening of COR proteins within the synaptonemal complex.

## Teaser

Meiotic crossovers are highly regulated between and along chromosomes, but the mechanisms that govern crossover patterning are hotly debated. One attractive proposal is that some protein initially has minor accumulations at all recombination sites, but through the biophysical process of coarsening, becomes enriched at one or a few sites per chromosome, while being depleted at other locations. Frantz *et al*. tested a prediction of this model in *Drosophila* by changing the dosages of these proteins. Crossover numbers rise and fall with increased and decreased dosage, which is consistent with a key prediction of the coarsening model.

## Introduction

Crossovers between homologous chromosomes facilitate reductional segregation in meiosis. Meiotic recombination is initiated by the introduction of DNA double-strand breaks (DSBs). A subset of these are repaired as crossovers, with the rest being repaired into noncrossover products. Crossover patterning processes (reviewed in Pazhayam et al. 2021) determine which sites are repaired to give crossovers. Perhaps the most important patterning outcome is crossover assurance, which dictates that each pair of homologs usually has at least one crossover per meiosis, regardless of chromosome size (Owen 1949). Crossover assurance is important because chiasmata resulting from crossovers provide physical linkages that promote stable biorientation of homologous chromosomes on the meiotic spindle, thereby promoting reductional segregation. Consequently, eliminating crossing over results in high rates of meiotic chromosome nondisjunction .

Another aspect of crossover patterning is interference, originally described by Sturtevant (1913) as the tendency of the *X* chromosomes in *Drosophila* to have only one crossover per meiosis. Subsequent definitions of interference include a significant reduction in double-crossovers (DCOs) in adjacent chromosome intervals compared to the number expected if the two intervals are independent of one another, or larger spacing between crossovers than expected in cases where there are two crossovers on one chromosome or chromosome arm (reviewed in Berchowitz and Copenhaver 2010; Otto and Payseur 2019). Although interference is widespread, occurring in plants, fungi, and metazoa, the reasons for its existence have been less clear.

**Fig. 1.**
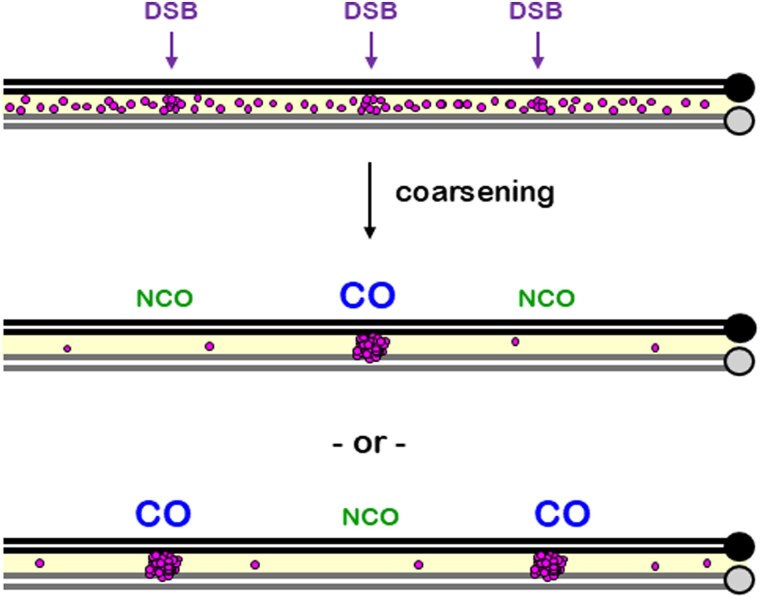
Illustration of the coarsening model. The drawing at the top represents a pair of synapsed homologous chromosomes (circles on the right ends are centromeres, lines are chromatids). Shaded background represents the synaptonemal complex; small circles are crossover-associated RING finger proteins. Early in pachytene, CORs are distributed throughout the SC, but are enriched at DSB sites. Coarsening results in a single large accumulation at one DSB site and a corresponding loss of CORs from the other sites (middle). The site with the accumulation is designated to becomes a crossover, whereas the other two are repaired into noncrossover products. Occasionally, two sites accumulate enough CORs to achieve crossover designation (bottom). This is only stable if the sites are far enough apart to prevent one taking CORs from the other. In this model, changes to the amount of CORs available might lead to changes in the number of crossover designations.

Recently, it was proposed that crossover assurance and interference result from the biophysical process of coarsening (Morgan et al. 2021; Zhang et al. 2025). In this model (Fig. 1), crossover designation factors are distributed within the synaptonemal complex, a protein structure that assembles between paired homologous chromosomes. Rog et al. (2017) presented observations and experiments that suggest the SC is phase-separated, and Zhang et al. (2018) proposed that crossover designation occurs by diffusion of proteins within the SC. In the coarsening model, these factors are initially distributed through the SC but are enriched at DSB sites (Fig. 1). Surface tension forces then drive the accumulation of crossover designation factors at one or a few sites per SC, with other sites becoming depleted for these factors. This model explains crossover assurance, provided an SC has sufficient crossover designation factor to make at least one accumulation. If there is enough of the designation factor(s), additional designations may occur along a single SC, but because nearby sites will exchange these factors until one becomes depleted, two accumulations will only happen if they are far apart. Thus, the coarsening model explains interference not as a phenomenon with a selective advantage, but as a consequence of the process that provides crossover assurance.

Crossover-associated RING finger proteins (CORs) exhibit behaviors consistent with them being crossover designation factors that accumulate through coarsening. These include HEI10 in *Arabidopsis thaliana* (Morgan et al. 2021) and ZHP-3 and ZHP-4 in *Caenorhabditis elegans* (Zhang et al. 2025). The *Drosophila melanogaster* genome encodes three CORs, named Vilya, Narya, and Nenya (Lake et al. 2015). Vilya physically interacts with Mei-P22, the noncatalytic subunit of the enzyme that generates meiotic DSBs, and is itself required for DSB formation. Studies of HA-tagged Vilya found that it is initially localized throughout the central region of the SC, but as meiosis progresses, Vilya becomes less intense along the SC and more intense at foci that are similar in number to the average number of crossovers per meiosis (Lake et al. 2015). The requirement for Vilya in making DSBs precluded direct genetic tests of whether it has a later function in designation or execution of crossing over.

A study of Narya and Nenya by Lake et al. (2019) concluded that the *narya* gene arose from a duplication of *nenya* in the ancestor to the melanogaster species subgroup, and that simultaneous loss of Narya and Nenya results in failure to make meiotic DSBs. An in-frame deletion allele of *narya* that is predicted to delete five residues, including the last cysteine of the RING finger domain, is competent to make DSBs, but not to make crossovers when *nenya* is simultaneously knocked down. Together, these data show that DSB formation requires Vilya plus either Narya or Nenya. The separation-of-function allele of *narya* shows that Narya or Nenya is required to make crossovers, and suggests that this function requires an intact RING domain, though it is unknown whether ubiquitin ligase activity is necessary.

The requirement of Drosophila CORs for DSB formation makes it difficult to use mutations to assess functions in crossover designation. However, if crossover designation occurs through coarsening of COR proteins, the number of crossovers would be predicted to be related to the amount of those proteins: Decreasing COR expression would result in fewer crossovers and increasing COR proteins might result in additional crossovers (if the system has the capacity to accommodate additional protein). To test this prediction, we measured crossing over in flies heterozygous for a deletion of *vilya* and in flies with an extra copy of *vilya* or coordinately overexpressing Vilya, Narya, and Nenya. Reducing Vilya dosage resulted in a significant decrease in the occurrence of double-crossover chromosomes, whereas the presence of an extra copy of *vilya* or coordinate overexpression of all three CORs led to a significant increase in crossovers. These results, together with previously published data, support a role for Vilya, Narya, and Nenya in meiotic crossover designation through coarsening.

## Materials and methods

### Drosophila genetics

Flies were raised on standard medium purchased from Archon Scientific (Durham, NC). Deletion and duplication stocks were obtained from the Bloomington Drosophila Stock Center. The deletion used to reduce *vilya* dosage was *Df(1)ED6630* (RRID:BDSC_8948), which removes a total of 41 genes. The duplication used was *Dp(1;3)DC406* (RRID:BDSC_31456), which duplicates 23 protein-coding genes in their entirety. These and other genetic elements are described in Flybase (release FB2025_05, Öztürk-Çolak et al. 2024).

### Transgene construction and transformation

The UAS.VNN transgene was constructed using GoldenBraid cloning (Sarrion-Perdigones et al. 2013; Matinyan et al. 2021). Vectors and some GoldenBraid parts were obtained from Addgene, and parts we made were deposited into Addgene; accession numbers are in parentheses. A transcription unit (TU) for each COR was assembled by combining a 10xUAS (252564), the hsp70z basal promoter (252565), the Vilya, Narya, or Nenya open reading frame (intronless coding sequences flanked by sequences for cloning into pUPD2 were synthesized as gBlocks by Integrated DNA Technologies, Inc.), and an HSV-tk terminator (165801). The Vilya and Narya TUs were assembled into Alpha1 (118044) to generate UAS.Vilya@A1 and UAS.Narya@A1. The Nenya TU was constructed in Alpha2 (118045) to generate UAS.Narya@A2. A $y^{+}$ transgene was cloned into Alpha2 to generate y@A2 (252566). UAS.Vilya@A1 was combined with y@A2 into Omega1 (118046), and UAS.Narya@A2 was combined with a *bsr* gene in Alpha2 (derived from 165,838) into Omega2 (118047). The resulting UAS.Vilya+y@O1 and UAS.Narya+bsr@O2 were combined back into Alpha1 to make UAS.Vilya+y+UAS.Narya+bsr@A1. This was combined with UAS.Nenya@A2 into Omega1.attB, which is an Omega1 vector into which we inserted a *phiC31 attB* site (252567). The resulting plasmid had *UAS::Vilya*, y+, *UAS::Narya*, *bsr*, *UAS::Nenya*, and the *attB* site. This plasmid was sent to GenetiVision (Houston, TX) for injection into VK31, a strain with a phiC31 *attP* insertion in 62E1. Adults that developed from injected embryos were crossed to *y w*; *TM3, Sb*/*TM6B, Tb Hu*, and progeny were screened for expression of the $y^{+}$ marker. Integrations were confirmed by PCR.

### Recombination and interference

To measure crossing over on *2L* we made females heterozygous for a chromosome carrying $dpp^{d-ho}$  *dpy* (unknown allele) and $Adc^{b-1}$ (referred to hereafter with the classical gene name *b*) and a chromosome carrying *P*{$w^{+}$}*KG03050*, which is inserted about midway between *dpy* and *b*. Virgin females heterozygous for these two chromosomes were backcrossed to $w^{1118}$; $dpp^{d-ho}$  *dpy*  $b^{1}$ males and the relevant phenotypes (heldout wings versus normal posture wings; dumpy wings versus normal shape wings; red eyes versus white eyes; and black body versus normal body color) were scored in the progeny. Genetic distances are expressed here using the more common centiMorgan (cM) unit rather than the equivalent map units traditionally used in *Drosophila*. Measures of interference were calculated using the equations of Stevens (1936). The coefficient of coincidence (*c*) is calculated as $\frac{\left(d)(n\right)}{\left(a)(b\right)}$, where *a* and *b* are the number of single-crossover progeny in the two intervals being compared, *d* is the number of double-crossover progeny, and *n* is the total number of progeny scored. This is equivalent to observed DCOs divided by expected DCOs if there is no interference. Interference (*I*) is equal to 1-*c*, and typically ranges from 0 (no interference) to 1 (complete positive interference, meaning no DCO progeny).

### Statistical analyses

For cM, *f* (crossover fraction) is the number of crossovers divided by total progeny scored (*n*). Variance (*V*) is *f*(1−*f*)/*n*, and standard deviation (SD) is $\sqrt{V}$. 95% confidence intervals were calculated as SD*1.96. For comparisons of crossover numbers in two genotypes, we used GraphPad QuickCalcs to conduct chi-square tests with Yate’s continuity correction. To compare interference values, we did Fisher’s exact tests using observed and expected number of DCOs for each genotype. For distributions of parental, single crossover, and double crossover classes, we did chi-square tests between observed and expected, where expected was the number expected in a given sample size if the distribution between classes was the same as in the control.

## Results and discussion

### Reducing vilya dosage leads to a reduction in double-crossovers

**Fig. 2.**
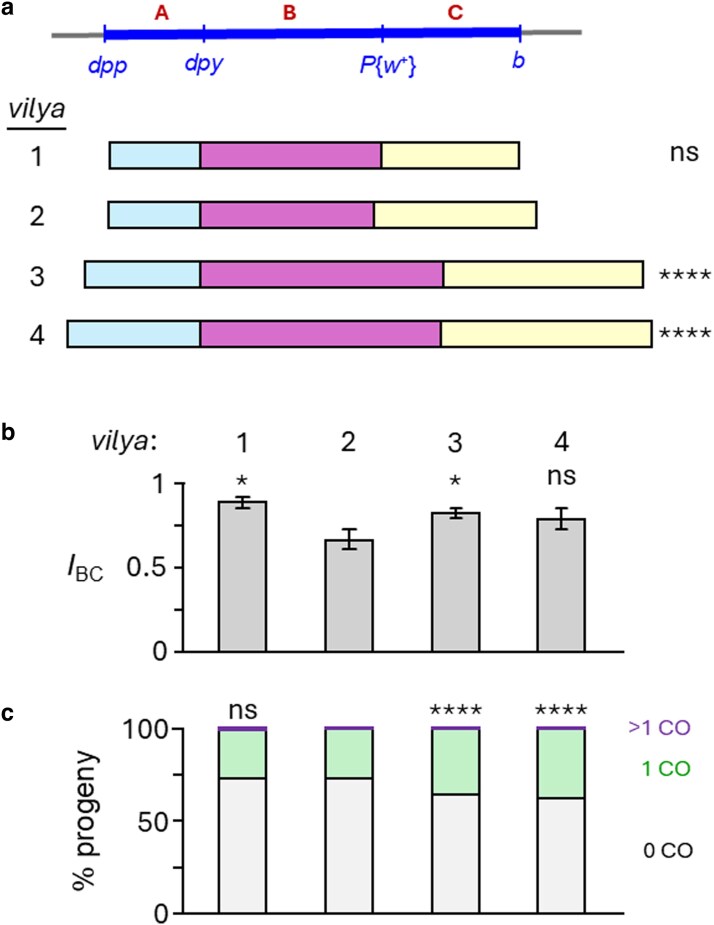
Effects of *vilya* dosage on recombination. a) The line at the top is a genetic map of *2L* The region in which crossovers were scored (thick bar) is divided into three intervals (a, b, and c). In a previous study using similar markers (Hatkevich et al. 2017), the genetic length of *2L* was 45.8 cM; the region scored here comprises more than 75% of the genetic length of this arm. Bars below show genetic lengths of each interval (A is blue, left; B is magenta, center; and C is yellow, right) in flies with one to four copies of *vilya*. Statistical significance is indicated for the sum of all three intervals compared to the wild type (two copies of *vilya*): ns: P>0.05; ****: P<0.0001. b) Interference values for different *vilya* dosages. Interference was calculated between the two larger intervals on the right. Error bars are 95% confidence intervals. Statistical comparisons are to flies with two copies of *vilya*. ns: P>0.05; *: P<0.05. c) Distributions of numbers of crossovers in progeny. Light gray (bottom), no crossovers; green (middle), one crossover; purple (top), two or three crossovers. Statistical significance is indicated for comparisons of the parental (no crossovers) class to wild type (two copies of *vilya*): ****: P<0.0001; ns: P>0.05. Counts of all categories are in Table 1.

To address whether reduced COR dosage impacts meiotic crossing over in Drosophila, we first assayed crossovers in females heterozygous for a deletion that removes *vilya*. In wild-type females, the genetic length of the interval assayed was 28.4 cM (Fig. 2a). In females heterozygous for a *vilya* deletion, the number of crossovers was not significantly changed (27.2 cM; 7,666 progeny; P=0.86). However, there were significantly fewer double-crossover chromosomes in the *vilya* deletion flies: 15 among 7,666 progeny, versus 64 among 6,805 progeny of wild-type females (P<0.0001; Table 1). We therefore calculated interference between the two larger, adjacent intervals (B and C). In Stevens’ measure of interference, *I*, where I=1 indicates complete interference (no DCOs) and I=0 means no interference (the number of DCOs observed is the same as the number expected if there is no interference). In the dataset from wild-type females (Table 1), we expected 48 DCOs if there was no interference; we observed only 29 (I=0.66±0.12; P<0.0001). In flies heterozygous for a deletion of *vilya*, we expected 89 DCOs if there was no interference but observed only nine (I=0.89±0.07; P<0.0001). This is a significant reduction in interference compared to flies with two copies of *vilya* (Fig. 2b, P=0.0282).

**Table 1.** Crossover data.

Genotype	P	SCOs			DCOs			TCOs	n	Total SCO	Total DCO	Total COs	cM A	cM B	cM C	Total cM
		A	B	C	AB	AC	BC									
1 vilya	5,591	456	915	689	2	4	9	0	7666	2060	15	2,150	6.03±0.05	12.08±0.07	9.16±0.07	27.26±0.10
2 vilya (wt)	4940	378	739	684	15	20	29	0	6,805	1,801	64	2,185	6.07±0.06	11.51±0.08	10.77±0.07	28.35±0.11
3 vilya	5,533	629	1371	1093	5	30	31	0	8692	3093	66	3489	7.64±0.06	16.19±0.08	13.28±0.07	37.10±0.10
4 vilya	1,175	158	292	248	2	8	9	0	1892	698	19	812	8.88±0.13	16.01±0.17	14.01±0.16	38.90±0.22
No driver	3,815	301	642	462	6	11	14	0	5251	1405	31	1591	6.06±0.07	12.61±0.09	9.27±0.08	27.94±0.12
bam::GAL4	3,414	298	643	507	6	10	31	2	4911	1448	47	1766	6.43±0.07	13.89±0.10	11.20±0.09	31.52±0.13
nos::GAL4	5,215	439	998	810	5	31	53	0	7551	2247	89	2781	6.29±0.06	13.98±0.08	11.84±0.07	32.11±0.11

The top four rows are from varying number of copies of *vilya*, and the bottom three rows are from the transgene overexpression of all three CORs. Columns on the left are counts of progeny whose genotypes were parental (P) or single, double, or triple crossover (SCO, DCO, TCO). Intervals A, B, and C are as in Fig. 2a. *n* is the sum of all flies scored. Total COs = SCO + 2*DCO + 3*TCO. cM columns indicate genetic distances and 95% confidence intervals.

The significant decrease in DCOs in females heterozygous for a *vilya* deletion is consistent with Vilya playing a role in crossover designation through coarsening, because reduced Vilya would lead to a corresponding reduction in the number of condensates that lead to crossover designation. Since *2L* comprises about 20% of the euchromatic genome, reducing Vilya by 50% may leave a sufficient quantity to still have an average of one crossover designation per meiosis on each synaptonemal complex, while significantly decreasing the occurrence of two designations on *2L*. In wild-type females, if each of the 64 double-crossovers was instead a single crossover, the reduction in number of crossovers would not be statistically significant for this sample size. Thus, a significant decrease in DCOs is not inconsistent with not detecting a significant decrease in total crossovers. The apparent increase in interference when one copy of *vilya* is removed may be a mathematical result of reduced ability to form two crossover-designating accumulations of CORs in the interval we assayed, rather than a change in the mechanism of interference *per se*.

### Crossovers are increased when there are additional copies of *vilya*

We also assayed the effects of increased Vilya dosage, using a duplication of *vilya* on an autosome. In females with one copy of the duplication, crossovers were significantly increased (37.1 cM in the region assayed, compared to 28.4 cM in wild-type females; P<0.0001). The intervals within which we measured crossovers do not cover all of *2L*. It is possible that the apparent increase represents a redistribution of crossovers from the ends of the arm into the region we assayed. It previous measurements across the entire arm (*e.g.* Hatkevich et al. 2017; Brady et al. 2018, the region we measured in these experiments comprises 77% of the total genetic length of *2L*. If the increased crossing over in *vilya* duplication flies was due to redistribution, it would require all of the crossovers on *2L* to be moved into the region we assayed. Although possible, we believe it more likely that there is an increase in crossovers across the chromosome.

In females homozygous for the duplication, and therefore carrying four copies of *vilya*, there was no additional increase in crossovers (38.9 cM versus 37.1 cM, P=0.15). We are unable to measure the amount of Vilya protein in pachytene cells, so it is possible that the lack of an additional increase in crossovers in flies homozygous for the duplication is due to feedback or protein degradation that limits overexpression, bounds on how much Vilya can exist within the synaptonemal complex, or the availability of the presumptive binding partners Narya and Nenya.

Overexpression of CORs might not be expected to affect the strength of interference, since nearby accumulations would still undergo exchange until there is only one. In our dataset from *vilya* duplication females, interference was modestly increased (I=0.834; P=0.0155). This may seem to be incongruent with increased crossing over. However, in wild-type females, 72.6% of the progeny had the parental configuration of markers and 26.5% had a chromosome with one crossover (Fig. 1c). In contrast, among the progeny of females with one copy of the duplication, 63.7% had no crossovers and 35.6% had a single crossover (P<0.001). Thus, the elevated crossing over when Vilya dosage in increased is due to more chromosomes with one crossover and fewer with no crossovers. Still, the apparent increase in interference in flies with one extra copy of *vilya* does not fit simple predictions of the coarsening model. Further studies may provide insight into whether this is a statistical anomaly, is due to an unknown genetic differences in the fly stocks used, or reveals an unknown feature of coarsening or roles of Vilya, Narya, and Nenya.

### Crossovers are increased when all three CORs are overexpressed

**Fig. 3.**
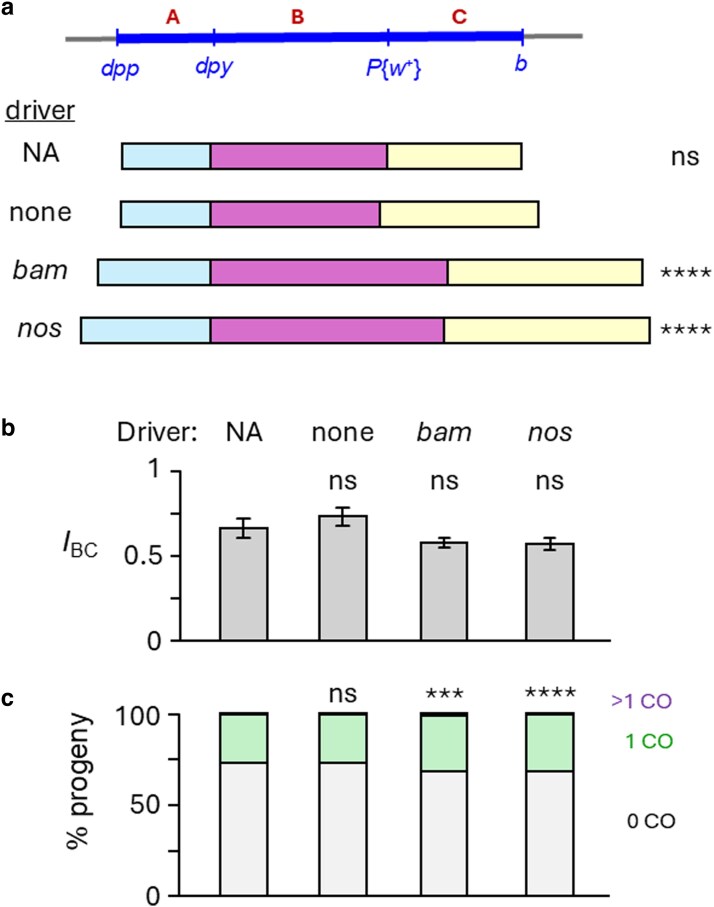
Crossing over when Vilya, Narya, and Nenya are coordinately overexpressed. Driver refers to Gal4 transgenes that drive expression of *UAS::vilya*, *UAS::narya*, and *UAS::nenya* NA: not applicable (control with no transgene, which are the data from Fig. 2 for flies with two copies of *vilya*); none: no Gal4 driver; bam: *bam::GAL4* driver; nos: *nos::GAL4* driver. Panels are as in Fig. 2, Statistical significance is in comparison to flies with no Gal4 driver.

*Caenorhabditis elegans* ZHP-3 and ZHP-4 interact physically (Nguyen et al. 2018; Zhang et al. 2018). In a yeast two-hybrid assay, Vilya, Narya, and Nenya each interact with themselves and with each other (Lake et al. 2019). We therefore tested the effects of coordinate overexpression of all three proteins. We constructed a fusion of a *10xUAS::hsp70z* promoter to sequences encoding each COR, then assembled a plasmid carrying all three, a $y^{+}$ gene as a marker for transformation, and a phiC31 *attB* site for integration into a genomic *attP* landing site. We scored crossovers in females carrying this transgene and either *bam::GAL4* or *nos::GAL4*, both of which express Gal4 in the female germline beginning before pachytene (Rørth 1998). The genetic length of the test interval increased from 28.4 cM in control females (no Gal4 expression) to 31.5 cM in those carrying the *bam::GAL4* transgene (P<0.0001) and to 32.1 cM in those with the *nos::GAL4* transgene (P<0.0001) (Fig. 3a). There was no significant change in interference, although there were significantly more double crossover chromosomes in both overexpression genotypes (P=0.03 for *bam::GAL4* and P=0.001 for *nos::GAL4*) and the only two triple crossovers chromosomes we saw were from the progeny of females with *bam::GAL4* (Table 1). As was the case for the *vilya* duplication, the increased crossovers in the *bam::GAL4* or *nos::GAL4* experiments are observed primarily as an increase in single-crossover chromosomes (Fig. 3c). We conclude that overexpression of the three COR proteins in meiosis results in a significant increase in single-crossover chromatids.

### Functions of CORs in Drosophila recombination

Previous research using a combination of mutations and RNAi knockdown indicated that the Drosophila CORs are required both for DSB formation and for crossover generation (Lake et al. 2015, 2019). Narya and Neyna appear to be redundant with one another these experiments. However, given that the gene duplication that generated *narya* occurred about 10–12 million years ago (Kumar et al. 2022), and both *narya* and *nenya* are apparently intact in all nine species in the melanogaster subgroup, it seems likely that each has some important unique function. Alternatively, expression of both might be required to achieve a sufficient concentration to ensure a least one crossover designation per major chromosome arm per meiosis.

Cytological studies show that Vilya is distributed through the SC but is enriched at sites of DSBs early in pachytene, then later is found at foci that are similar in number per nucleus to the number of crossovers, and are typically one per arm (Lake et al. 2015, 2019). These observations are consistent with the coarsening model. Our findings described above provide further support this model. Decreasing COR dosage would be expected to decrease the number crossovers, which we detected as a reduction in double-crossover chromosomes. Conversely, if the system can accommodate increases in COR proteins, then overexpression would cause increased crossing over. We observed significant increases in crossover number in flies with a *vilya* duplication and in flies overexpressing all three CORs.

Crossover designation through coarsening may help to explain some interesting observations in *Drosophila* recombination. One is that the small chromosome *4* never has crossovers in wild-type females (reviewed in Hartmann and Sekelsky 2017). Based on the occurrence of crossovers in mutants that lack Blm helicase, Hatkevich et al. (2017) proposed that meiotic DSBs are made on this chromosome, though the crossovers in the *Blm* mutant were proposed result from mitotic-like repair. If meiotic crossover designation requires accumulation of some minimal amount of CORs, it may be that the SC of *4* is too short to harbor enough CORs to make such an accumulation. It might be possible to overcome this limitation by overexpression, but given that the other chromosome arms are each more than five times the physical length of *4* and each averages just over one crossover per meiosis, it seems doubtful that the relatively short SC of *4* could accommodate enough CORs to permit a crossover designation.

Another interesting observation is that crossover numbers can vary significantly different between closely related species or even among different populations of one species (reviewed in Johnston 2024). For example, *Drosophila mauritiana*, a sibling species to *D. melanogaster*, has almost twice as many crossovers per meiosis as *D. melanogaster* (True et al. 1996). Brand et al. (2018) showed that some of this increase can be explained by rapid evolution of the *mei-218* recombination gene. We speculate that variation in COR expression or interactions between CORs and synaptonemal complex proteins might also account for some of the differences in crossover number between populations or between closely related species.

## Data Availability

All raw data are in Table 1. Drosophila strains and plasmids not deposited into AddGene are available upon request.
